# Development of an Objective Index for Evaluating New Fining Agents Used in Winemaking: A Case Study of the Cell Wall Material from Red Grape Skin

**DOI:** 10.3390/foods14213708

**Published:** 2025-10-30

**Authors:** Julia Gómez-Pérez, Berta Baca-Bocanegra, M.ª Lourdes González-Miret, José Miguel Hernández-Hierro, Julio Nogales-Bueno

**Affiliations:** 1 Food Colour and Quality Laboratory, Área de Nutrición y Bromatología, Facultad de Farmacia, Universidad de Sevilla, 41012 Sevilla, Spain; jgomez11@us.es (J.G.-P.); miret@us.es (M.L.G.-M.); 2 Department of Analytical Chemistry, Facultad de Farmacia, Universidad de Sevilla, 41012 Sevilla, Spain; bbaca1@us.es (B.B.-B.); julionogales@us.es (J.N.-B.)

**Keywords:** phenolic composition, fining agents, red wine, anthocyanins, flavanols, turbidity

## Abstract

This study aimed to establish a fining index based on objective criteria to evaluate and compare the effects of various commercial fining agents on red wine. The agents included pea and potato protein isolates, porcine gelatine, and bentonite. The phenolic composition of Syrah wine was analysed to assess the impact of clarification on anthocyanins, flavanols, flavonols, and overall turbidity. Results showed significant variability among agents, with gelatine causing the highest phenolic reduction (%) and fining efficiency, reducing flavanols by 33.80%. In contrast, bentonite showed minimal impact on phenolic content, with a reduction of only 0.02% in flavanols. A fining index was proposed, allowing for the classification and prediction of untested fining agents’ effects based on similarity to known agents. This index could facilitate more precise selection of fining agents, optimizing wine quality while addressing allergenicity and fining concerns. Moreover, its application could help manufacturers reduce production costs by selecting the most efficient fining alternatives. Furthermore, a case study of the cell wall material from red grape skin was evaluated using samples from three different physiological states.

## 1. Introduction

Wine clarity is an attribute that is increasingly valued by consumers, making it a relevant criterion for producers. The presence of suspended particles is often perceived as an indication of poor quality, even though it does not necessarily affect the wine’s organoleptic properties. The fining process is commonly associated with the removal of turbidity but strictly refers to enhancing wine transparency [1]. Unfined wines are generally considered to be turbid, which can result in them being perceived as lower quality [2], even though some contain insoluble compounds that do not affect sensory acceptability [3]. Turbidity in wine is caused by suspended particles that scatter light, producing the Tyndall effect. This effect enables turbidity to be visualised and quantified, providing a basis for assessing wine clarity based on particle concentration and size [4]. Two types of fining in wine can be distinguished: natural clarification and fining with fining agents.

Natural clarification occurs during the winemaking process, leading to the precipitation of particles in suspension. However, this process is typically insufficient, as the remaining unstable compounds can interact with pigments and colourless phenolic compounds, altering the sensory characteristics of the wine and thereby affecting its overall quality [2,5].

Fining is a common practice in winemaking aimed at enhancing the stability of wine by removing or reducing components that contribute to undesirable qualities and that may also pose a risk to the food safety of the final wine [5,6,7,8]. This is achieved by introducing a reactive or adsorptive material [5,9,10]. The interaction between the fining agent and phenolic compounds influences the wine’s colour, as pigments are precipitated [11,12], while interactions with tannins result in changes to the wine’s astringency and bitterness [13,14]. These fining agents can have either positive or negative effects depending on the quantity and type of agent selected. Proper fining can result in smoother, more balanced wines, while improper fining treatments may lead to wines that are less harmonious or, in other words, an undesired reduction in colour [15].

Numerous fining agents, particularly those of proteinaceous origin, are used in winemaking because proteins have a strong ability to bind with phenolic compounds [16,17]. Traditionally, proteins such as gelatine, egg albumin, casein and isinglass have been used for fining tannin compounds, due to their high affinity for proanthocyanidins. However, these proteins are of animal origin and may trigger allergic reactions [18,19,20,21] in sensitised individuals, leading to dermatological, respiratory or gastrointestinal symptoms [22,23,24]. On the other hand, with the rise of new trends (vegetarian or vegan), there has been an increased demand for wines elaborated without fining agents from animal sources [18]. Plant-derived agents are offering promising alternatives, particularly for vegan wines, by achieving reductions in specific phenolic compounds comparable to those of animal-derived agents. Consequently, the market has seen the introduction of agents derived from cereal proteins, legumes (such as peas), and tubers (such as potatoes) [21].

In this regard, the International Organization of Vine and Wine (OIV) authorized the use of plant proteins derived from wheat and peas in resolution OENO 28/2004 [18,19]. It is also important to note that EU Directive 2007/68/EC (27 November 2007) indicates the legal obligation to indicate their presence on the label. In 2012, the European Union, to manage this risk, introduced a regulation stating that egg and milk proteins must be declared on wine labels if they are found in wine at a concentration greater than 0.25 mg/L [18,19]. All these facts have led to the search for new fining agents.

Given the large number of fining agents currently available on the market, it is important to consider that their characteristics, such as type, degree of hydrolysis, dosage, and contact time, play a fundamental role in the final attributes of the wine [2,10,13,14,25,26]. Additionally, factors directly related to the wine, such as its type, age, composition, and the chemical properties of the matrix, also influence the final product [2,20,26,27].

Thus, the main objective of this study was therefore to define a numerical index for classifying fining agents according to their fining capacity. To this end, the index was tested using cell wall material from red grape skins at various stages of ripeness. The effectiveness of various commercial fining agents in reducing turbidity and phenolic compounds (such as anthocyanins, flavanols and flavonols) in Syrah wines from warm climates was evaluated to develop this index.

## 2. Materials and Methods

### 2.1. Fining Agents

Fining agents used in the fining trials were porcine-derived gelatine (Vinigel forte) and bentonite (Volclay), as they are two of the most employed fining agents in the winemaking industry. Additionally, commercial powdered protein isolates from pea (Proveget 100, *Pisum sativum* L.) and potato (Proveget fine, *Solanum tuberosum* L.) were used. The fining agents were provided by Agrovin S.A. (Ciudad Real, Spain) and approved by the International Oenological Codex and EC 606/2009 regulation.

Furthermore, three cell wall samples extracted from Tempranillo grape skins were used as fining agents. These samples were collected from a local vineyard in Lleida (Spain) at three different stages of Tempranillo grape ripening: pre-harvest (P), harvest (H) and over-ripening (O). Samples from the pre-harvest stage were taken 22 days before harvest, and samples from the over-ripening stage were taken 19 days after harvest. The process of obtaining and chemical composition of the samples is detailed in the original article [28]. The efficacy of the three cell walls as fining agents was tested using the proposed index.

### 2.2. Winemaking Process and Fining Treatments

Red wine used in this study was produced from *Vitis vinifera* L. var. Syrah, sourced from the region of Condado de Huelva Designation of Origin (D.O.) located in the southern part of Spain, specifically in Andalusia, where a warm climate predominates. This red grape is a resistant cultivar to warm climatic conditions and to produce high-quality red wines [29]. Grapes were harvested at maturity stage (13.1 °Be, pH 3.52, and 4.80 g/L as tartaric acid). The characteristics of the base Syrah wine, determined according to the official methods described by the OIV [30], were: pH = 4.16; alcohol content = 13.7% *v*/*v*; reducing sugars = 1.43 g/L; volatile acidity = 0.29 g/L as acetic acid; free sulphur dioxide = 46 mg/L; total sulphur dioxide = 60 mg/L; turbidity = 6.89 NTU (Nephelometric Turbidity Unit); total phenolic content = 3378.69 mg/L; flavanol content = 380.92 mg/L; flavonol content = 113.12 mg/L; and anthocyanin content = 233.88 mg/L. The wine was produced during the 2023 harvest season in an experimental-scale winery, using stainless steel tanks, following by traditional vinification methods. Fining processes were carried out just after the end of malolactic fermentation. The fining assays were performed in triplicate, in 200 mL glass containers, for each fining agents, which were prepared following the recommendation of manufacturers. The doses applied of each product were the maximum recommended (0.1 g/L).

For each treatment, the required amount of fining agent was weighed and dissolved in a small volume of wine. Once fully dissolved, the solution was returned to the corresponding 200 mL container. After addition, the wine was homogenised using a glass rod to ensure the fining agent was distributed uniformly. The containers were then sealed and kept at room temperature (25 ± 2 °C) in the absence of light for 6 days (standard mean conditions for the normal treatment in wineries). Moreover, triplicates of 200 mL of wine without fining agent were used as control wine. After 6 days of fining, wine samples were separated from lees.

### 2.3. Analysis of Wine Anthocyanins and Flavonols by HPLC

To measure the anthocyanin and flavonol contents, the wine was diluted 1:2 with 0.1 M HCl, filtered through 0.45 μm pore size filters and directly injected into the chromatographic system. The chromatographic analysis was carried out following a modification of García-Marino et al. [31] as described elsewhere in Hernández-Hierro et al. [32]. An Agilent 1200 (Palo Alto, CA, USA), equipped with quaternary pump, UV-Vis diode-array detector, automatic injector and the Chem Station software (B.04.03 version) (Palo Alto, CA, USA) was used for the analyses. Phenolic compounds were separated on a Zorbax C18 column (4.6 mm × 250 mm, 4.6 μm particle size) (Palo Alto, CA, USA) thermostated at 35 °C. Solvents were 0.1% trifluoroacetic acid (A) and 100% HPLC grade acetonitrile (B). The elution profile was as follows: 10% B for 3.25 min, from 10 to 15% B for 12.37 min, 15% B for 5.21 min, from 15 to 18% B for 5.21 min, from 18 to 30% B for 20.84 min, and from 30 to 35% B for 5.20 min. The flow rate was 0.8 mL/min, and the injection volume was 100 μL. The preferred detection wavelength was 520 nm for anthocyanins and 360 nm for flavonols. All analyses were performed in duplicate. All the results were expressed as mg of malvidin-3-*O*-glucoside (Sigma-Aldrich, Madrid, Spain) and mg of quercetin-3-*O*-glucoside (Sigma-Aldrich, Madrid, Spain) equivalents per mg/L of wine.

### 2.4. Total Phenol and Flavanol Analysis

Total phenolic content was determined using the Folin–Ciocalteu method [33]. Two hundred microliters of sample (1:40, wine:water) were mixed with 200 μL of sodium carbonate (20% *w*/*v*), 100 μL of Folin reagent and made up to 2 mL with ultrapure water. After a two-hour period, the absorbances of these solutions were measured against the blank (prepared in the same way with ultrapure water) at a wavelength of 765 nm using a cuvette with 1 cm optical path. Gallic acid (Sigma-Aldrich, Madrid, Spain) was used as a standard for construction of the calibration curve, and the concentration of total phenols was expressed as gallic acid equivalents in mg/L of wine.

Flavanol content was determined following a modification of Vivas et al. [34]. Ten microlitres of the 1:4 diluted wine was mixed with 190 μL of methanol and 1 mL of 4-dimethylaminocinnamaldehyde (DMACA) reagent. The DMACA reagent was prepared immediately before use, containing 0.1% (*w*/*v*) DMACA in a mixture of HCl:methanol (1:10, *v*/*v*). The absorbance was recorded at 640 nm after 10 min of reaction. A calibration curve of (+)-catechin (Sigma-Aldrich, St. Louis, MI, USA) was used for quantification and all the measures were within the linear range of the calibration curve. All the results were expressed as mg of catechin equivalents per mg/L of wine (Sigma-Aldrich, Madrid, Spain). Both Folin and DMACA analyses were performed on an Agilent 8453 UV–visible spectrophotometer (Palo Alto, CA, USA), equipped with diode array detection (DAD).

### 2.5. Analysis of Turbidity

Turbidity of wines was measured using a HI 83749 portable turbidimeter (Hanna Instruments, Woonsocket, RI, USA, EE.UU.) after 6 days of fining. Results were expressed in NTU (Nephelometric Turbidity Unit).

### 2.6. Statistical Analysis

Different statistical strategies were employed to study the effect of the fining agents on the composition of the phenolic families under evaluation. An analysis of variance (ANOVA) followed by Tukey’s post hoc test was performed to examine the influence of the fining agents on the phenolic composition of the wines. The percentages by which the calculated parameters were reduced were considered the dependent variables and the fining agent used was considered the independent variable. Additionally, hierarchical clustering (HC) analysis was performed to visualise the relationships among samples based on the content of the phenolic groups considered [35,36,37]. This clustering algorithm groups similar objects into clusters [36,38] and was selected because it performs well with small datasets, providing a complementary perspective to the ANOVA results. The analysis was based on squared Euclidean distances between the corresponding values of the different samples, and Ward’s linkage method was applied to organise and classify the parameters into distinct groups, as represented in the dendrogram. All statistical analyses were performed using Statistica v.8.0 software (StatSoft Inc., Tulsa, OK, USA, 2007).

## 3. Results and Discussion

### 3.1. Fining Test with Conventional Agents

In the following sections, the impact of the four commercial fining agents (bentonite, pea protein, potato protein and porcine gelatine) on the different phenolic families studied and on wine turbidity will be described.

#### 3.1.1. The Effect of Fining on Total Phenolic Content

All fined wines showed a significant reduction in total phenolic content. In this study, fining with porcine gelatine (Vinigel forte) resulted in the highest reduction percentage, with a decrease of 13.61% compared to the control wine (Table 1), a value of the same order as those reported in other studies [39,40,41]. This reduction can be attributed to the fact that phenolic compounds are negatively charged, which causes them to interact more actively with porcine gelatine (Vinigel forte), leading to the formation of floccules and thus increased sedimentation [41].

For wines fined with plant-derived proteins, a reduction of 2.92% was observed for potato protein compared to the control wine, showing a reduction similar to that obtained by Kang et al. [26]. Meanwhile, fining with pea protein (VF) resulted in a 6.66% decrease in phenolic compounds compared to the control wine, which represents a greater reduction than in the previously referenced study by Kang et al. [26]. In other studies, pea proteins at 0.40 g/L reduced total phenolics by 4.48%, similar to the reduction obtained in this study at a dose of 0.1 g/L, and in both cases, this was less than previously reported reductions of 9.04% of phenolics. Different fractions of pea proteins might explain this difference as well [42]. These variations between studies can be explained by differences in the composition and matrix of the wine itself. These factors include ethanol content, pH, ionic strength and initial phenolic concentration. These parameters directly influence protein-phenol interactions and the selectivity of the clarification process. Furthermore, it has been suggested that the denaturation or partial hydrolysis of plant proteins could modify their adsorption capacity, thereby altering the effectiveness and selectivity of the clarification process towards certain phenolic subclasses [14,43].

Similarly, our results align with those of Gambuti, Rinaldi, and Moio [42], Tschiersch et al. [14], and Gazzola et al. [19], who demonstrated that potato and pea proteins are good plant-based alternatives to animal proteins for reducing the content of compounds primarily responsible for astringency in wines, such as flavanols. This confirms the increasing potential of plant-derived fining agents to achieve adequate clarification while preserving desirable sensory attributes, particularly colour intensity and mouthfeel balance, thereby supporting the growing trend toward vegan-friendly winemaking.

In the case of bentonite, the reduction percentages are generally low compared with other fining agents. This is consistent with its mechanism of action, whereby it adsorbs positively charged proteins rather than phenolic compounds. As a result, bentonite shows little affinity for polyphenols and tends to preserve the wine’s phenolic composition. However, excessive use may still cause partial loss of colour and aroma, as some pigments and volatile molecules can also be adsorbed [44]. Overall, no significant differences were observed among the evaluated fining agents for the studied phenolic family, indicating that the fining process has a limited effect on total phenolic content under these conditions, achieving a good balance between clarification efficiency and phenolic preservation.

#### 3.1.2. The Effect of Fining on Anthocyanin and Flavonol Content

Regarding the effect on anthocyanins (Table 1), all samples exhibited a reduction in total anthocyanin content following the addition of commercial fining agents, with reductions ranging from 1.87% to 4.02%. This reduction is a normal consequence of the fining process, whereby anthocyanins (responsible for the red colour of the wine) can interact electrostatically or through hydrogen bonding with the fining agents, particularly proteins. These interactions lead to the partial precipitation or adsorption of the anthocyanins, which are then removed from the wine along with the fining agent. The highest anthocyanin reduction was observed with potato protein (4.02%), while the lowest reduction was observed with pea protein (1.87%).

Comparing the data from this study with previous research [42] shows that, at the same dosage, potato protein remains the most effective fining agent, although its effectiveness was reduced by 12.57% in that study. This difference may be due to variations in the composition of the wine, such as its pH level, ethanol content and the natural concentration and structure of its phenolic compounds. All of these factors affect the interaction between fining agents and anthocyanins.

In the case of flavonols, it is observed that the most effective fining agent is potato protein, reducing them by 4.22%, while bentonite is the least effective, with a reduction of 0.26%. For the two families studied in this section, after conducting the ANOVA, it is evident that there are no significant differences between the families when comparing the different fining agents. This suggests that, despite small numerical variations, the impact of the tested fining agents on these phenolic compounds is statistically comparable.

#### 3.1.3. The Effect of Fining on Flavanol Content

Flavanols are compounds that contribute to the tannin content (astringency) of wines, a sensory characteristic that is not always well accepted by consumers when present in excess. Reducing this gustatory sensation is the primary goal when fining a wine. Table 1 shows that the different fining agents mainly act on the removal of flavanols, with the most effective being Vinigel forte (porcine gelatine), showing a reduction of 33.80%. The other fining agents have a similar effect, except for bentonite. For the mineral fining agent (bentonite), the reduction in flavanols (astringency) is 0.02%, which can be considered insignificant. This suggests that the fining process did not affect astringency.

Granato et al. [45] reported that pea protein did not affect the flavanol composition of wine, indicating that this fining agent does not cause significant changes in these compounds. This result differs from the observations made in the current study, perhaps due to differences in the origin of the wines used for the fining trials between the studies. Table 1 shows that the phenolic compounds that undergo the greatest reduction after the fining stage are the flavanols. In the case of this family, significant differences can be observed between the fining agents used, finding two groups among them. It is observed that gelatine and bentonite produce significant differences in the percentages of flavanol reduction, while vegetable proteins show an intermediate effect.

#### 3.1.4. Impact on Wine Turbidity

It is crucial to assess wine turbidity as it determines whether the wine is suitable for bottling. At the time of bottling, the wine should exhibit an NTU value of ≤1; if the value exceeds this threshold, the wine requires further filtration stages [5].

This parameter has been studied independently because turbidity reduction is primarily associated with the removal of yeast, plant residues, and proteins, with minimal impact on the phenolic compound content [41,46].

The turbidity value (Nephelometric Turbidity Units, NTU) of Syrah wine after the fining process without any fining agents (control), once 6 days had passed, was 6.89 ± 1.17 NTU.

In comparison (Table 2), all fining agents showed a considerable fining effect, except for pea proteins, which caused an increase in turbidity with values similar to those reported in previous studies. As can be seen in the table, significant differences were found in the turbidity reduction percentage between the different fining agents. The increase in turbidity observed with pea proteins could be due to the formation of unstable aggregates or colloidal turbidity, possibly caused by poor interaction or lack of compatibility between the fining agent and the wine matrix. This behaviour has been observed in other studies when fining agents fail to effectively precipitate suspended particles [19]. Porcine-derived gelatine was the most effective agent, achieving a 50.25% reduction in turbidity compared to the control wine. The best clarifying agent was porcine-derived gelatine, which reduced turbidity by 50.25% respect to control wine. In the case of turbidity, significant differences can be observed between the different fining agents studied.

### 3.2. Development of Fining Index

**Figure 1:**
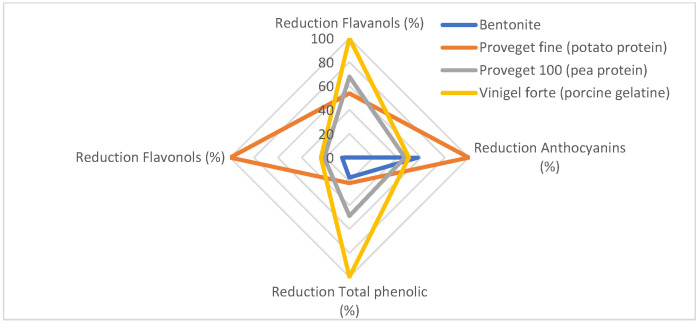
Radial chart showing the different reduction percentages after they have been normalized.

Before developing the fining index, a radial chart (Figure 1) was created to better compare the effects of the fining agents on the different compounds studied. To achieve this, the reduction percentages of the various compounds were normalized, as they have different magnitudes, making it difficult to evaluate the differences without this normalization.

As shown in the graph, bentonite mainly reduced anthocyanins, while the concentrations of the other phenolic compounds remained virtually unchanged. In the case of porcine gelatine, it is seen that the highest reduction is in flavanols with an average reduction in anthocyanins. Finally, vegetable proteins are the most similar to each other and similar in the reduction of flavanols to porcine gelatine.

**Figure 2:**
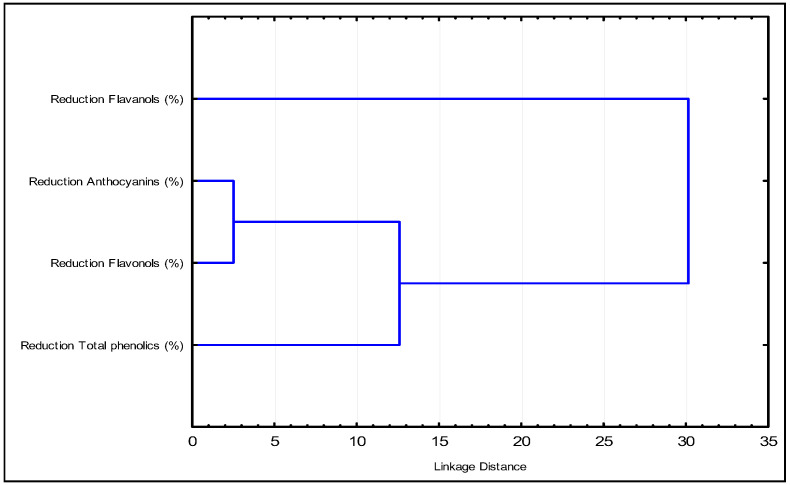
The dendrogram shows the grouping of the samples according to the effect on the content of the phenolic groups considered.

In the dendrogram (Figure 2), it is evident that anthocyanins and flavonols exhibit the smallest distance, indicating a similar reduction. As the Euclidean distance increases, total phenols join the group of anthocyanins and flavonols, while flavanols remain as a separate family. This suggests that flavanols are the compounds that predominantly influence the effect of the fining agents.

Then, based on the reduction values of the different families studied (Table 1), a fining index is proposed to evaluate other fining agents available on the market or new ones, with the aim of predicting their behaviour under similar conditions. In other words, the fining index denotes the degree of similarity between a target fining agent and one of the fining agents studied in this work. The closer the result of the index is to 100, the greater the similarity with the fining agent being studied. Below, Equation (1) developed for the fining index proposed in this study can be observed:

(1) $$IAi=\;100\;-\sqrt{{\left(∆Flavanol\right)}^{2}\;+\;{\left(∆Anthocyanins\right)}^{2}\;+\;{\left(∆Total\;phenolic\right)}^{2}\;+{\left(∆Flavonol\right)}^{2}\;}$$

where

• **IA*****i***: fining index for each of the fining agents studied;
• **∆Flavanols** = %R. Flava_x_ − %R. Flava*_i_*_;_
• **∆Anthocyanins** = %R. An_x_ − %R. An*_i_*_;_
• **∆Total phenolics** = %R. Tp_x_ − %R. Tp*_i_*_;_
• **∆Flavonols** = %R. Flavo_x_ − %R. Flavo*_i_*_;_
• **x** = target fining agent;
• ***i*** = studied fining agent (bentonite, pea proteins, potato proteins, and gelatine);
• **%R. Flava** = percentage reduction of flavanols;
• **%R. An** = percentage reduction of anthocyanins;
• **%R. Tp** = percentage reduction of total phenolics;
• **%R. Flavo** = percentage reduction of flavonols.

### 3.3. A Case Study of the Cell Wall Material from Red Grape Skin

In this study, the behaviour of three cell wall materials from red grape skin were analysed as potential fining agents to determine their similarity to a commercial fining agent.

Once the fining index has been established, a fining trial is conducted using the same wine and, as fining agents, three experimental fining agents consisting of the cell wall material from red grape skin [28], under the same conditions as the initial trial proposed in this study.

**Figure 3:**
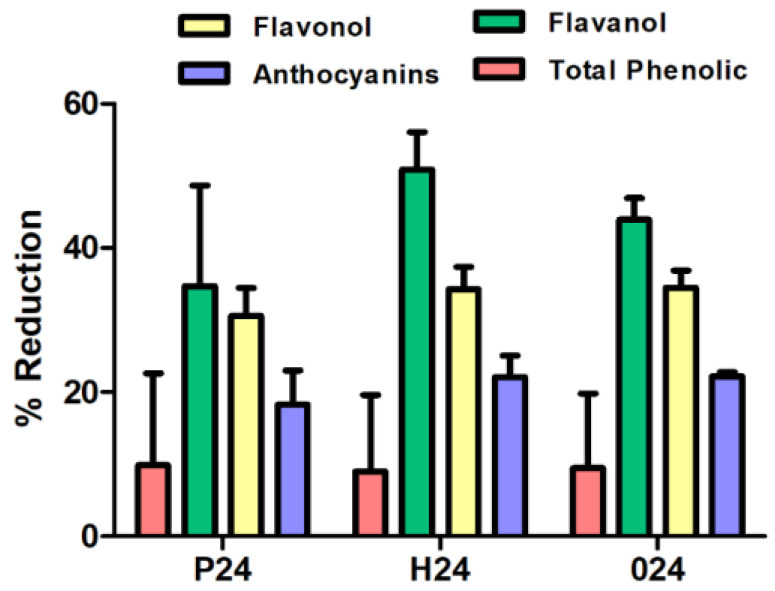
Column chart that presents the percentage reduction of phenolic compounds using cell wall material at three ripening stages: pre-harvest (P), harvest (H), and post-harvest (O).

In general, similar reduction percentages are obtained across the three ripening stages (Figure 3). The highest reduction percentages were observed for flavanols, corroborating previous findings with commercial fining agents and confirming that flavanols are the compounds most susceptible to reduction during the fining process. Among the three ripening stages, the harvest stage shows the highest flavanol retention, with 50.87%, followed by stage post-harvest and pre-harvest with retention percentages of 43.94% and 34.73%, respectively. Meanwhile, pre-harvest, harvest, and over-ripening cell wall samples showed a higher anthocyanin retention capacity compared to the commercial agents and were similar among each other.

Based on the fining index, Equation (1), that has been established and discussed in the previous section, the index is calculated for the three cell wall samples in order to see which commercial agent used in this study most closely resembles them (Table 3).

After calculating the fining index of the three cell wall samples, it was observed that they show the highest degree of similarity with the animal-based fining agent (Vinigel forte), ranging from 56.2 to 62.4. However, no significant differences were found with respect to the two plant proteins (pea and potato protein). Conversely, the samples showed a marked difference in similarity with the mineral-based fining agent (bentonite), ranging from 34.9 to 48.7. Although the samples share certain similarities with porcine gelatine, they show a notable reduction in anthocyanin content, which is not typically desired when selecting a fining agent. This suggests that, overall, the three samples exhibit unique behaviour, clearly distinguishing themselves from the most used commercial fining agents, as around 50% of their behaviour cannot be compared to that of these commercial agents.

For the pre-harvest sample, Tukey’s test shows that, although it is more similar to porcine gelatine in numerical terms, there are no significant differences compared to the other fining agents evaluated. For the harvest and post-harvest samples, a similar numerical trend towards porcine gelatine is observed. However, significant differences were only found with bentonite; no differences were observed with the two plant proteins.

**Figure 4:**
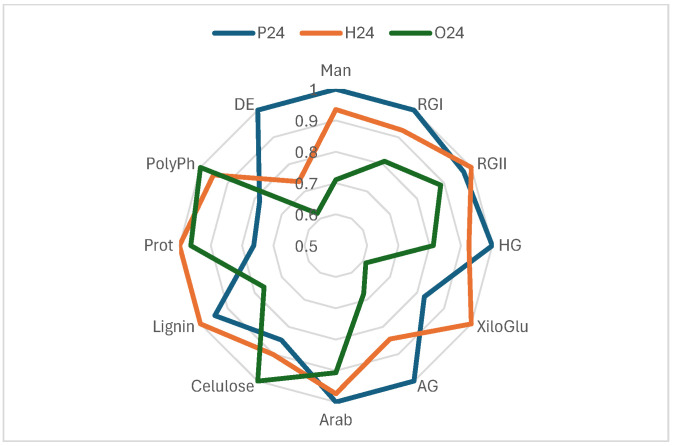
Radial graph comparing the composition based on the normalised values of three samples: P24, H24 and O24. Man (mannans), RGI (rhamnogalacturonan I), RGII (rhamnogalacturonan II**),** HG (homogalacturonan), XiloGlu (xiloglucans), AG (arabinogalactan), Arab (arabinans), Cellulose, Lignin, Prot (proteins), PolyPh (polyphenols), and DE (degree of esterification). Data from [28].

The effectiveness of cell wall materials from red grape skins, extracted at three ripening stages (pre-harvest, harvest, and post-harvest), was evaluated based on their chemical composition [28] and their ability to reduce phenolic compound content in wine. These materials were applied as fining agents, and their impact on total phenols, flavanols, flavonols, and anthocyanins was assessed (Figure 3). The compositional profiles, normalized and represented in radar charts (Figure 4), provide key insights into the structural and chemical properties that influence their fining performance.

Sample P24, corresponding to the pre-harvest stage, exhibited the highest concentrations of pectic polysaccharides (HG, RGI, AG, and Man) and the highest degree of esterification (DE), indicating a structurally intact and highly organized cell wall. However, it showed low levels of proteins and polyphenols, suggesting a simpler chemical composition. Consequently, its fining capacity was moderate, with reductions of 9.88% in total phenols, 18.25% in anthocyanins, 34.73% in flavanols and 30.58% in flavonols. These findings suggest that sample P24 may be a suitable fining alternative for wines where a limited reduction of phenolic content and preservation of colour are desired.

In contrast, sample H24, collected at technological maturity, the composition appears more balanced. Although its pectin content was slightly lower than that of sample P24, it had higher levels of proteins, lignin, and polyphenols, suggesting a more balanced cell wall matrix. This structural balance may explain its stronger fining performance, particularly regarding the removal of flavanols (50.87%) and flavonols (34.26%), along with a significant reduction in anthocyanins (22.10%). H24 appears to act as a more versatile and effective fining agent than the other two. Its ability to reduce astringent and bitter compounds with a moderate impact on wine colour positions it as a promising natural alternative to commercial fining products.

For its part, sample O24, derived from overripe skins, presented a profile with high levels of cellulose, arabinose, proteins, and polyphenols, and a very low content of pectins and degree of esterification. This structural degradation allowed strong interactions with the phenolic compounds in the wine, resulting in a significant reduction in anthocyanins (22.22%) and flavonols (34.55%), although it was slightly less effective than H24 in reducing flavanols (43.94%). However, the high polyphenol and protein content of O24 may pose a potential risk of unwanted compound transfer. Its use may be more appropriate in wines with a high phenolic load or those requiring intensive fining interventions.

The turbidity reduction showed a marked variation among the different ripening stages. At pre-harvest (P24), the highest reduction was observed (27.45%), while at harvest (H24) the reduction decreased to 17.31%. In the post-harvest stage (O24), the reduction increased again to 24.74%, suggesting a recovery in clarification capacity after harvest.

These results highlight the potential of using cell wall materials selected according to their state of maturation as natural and sustainable fining agents, with different levels of intensity and specificity depending on their composition.

## 4. Conclusions

This study reveals that the effectiveness of different commercial fining agents in modifying the phenolic composition of red wines varies according to the type of phenolic compound. Flavanols are particularly susceptible to modification. Porcine protein-based agents caused the greatest reduction in flavanols and total phenols, significantly altering the wine’s phenolic profile. Plant-based agents, such as pea and potato proteins, also notably reduce flavanols and offer a viable alternative to animal-based agents. By contrast, bentonite has a minimal impact on the phenolic profile, though it does reduce turbidity, albeit less effectively than porcine gelatine.

Plant-based proteins behave similarly to animal-based ones, whereas bentonite acts independently, reflecting the differences in their respective mechanisms of action. Flavanols behave independently of other phenolic families, while flavonols, anthocyanins and total phenols decrease proportionally. Based on these results, a fining index is proposed to classify and predict the behaviour of unknown agents according to their similarity to those studied. This provides a practical tool for oenological selection. It would be advisable for future research to explore its applicability in wines with other fining needs, such as white, sparkling, or rosé wines, in order to broaden its relevance and utility. Furthermore, given the promising results of using grape skin cell walls to reduce flavanols, it would be recommended to expand research in this area by exploring other sources of grape skin, such as white grape pomace, a by-product of the wine industry, or other by-products from the food industry that may behave as fining agents.

## Figures and Tables

**Table 1:** Percentages of *reduction of the* different families with respect to the control wine.

Fining Agents	Total Phenolic Content (%)	Flavanol Content (%)	Flavonol Content (%)	Anthocyanin Content (%)
Bentonite	2.28 ^a,A^ ± 16.21	0.02 ^a,A^ ± 35.78	0.26 ^a,A^ ± 1.32	2.31 ^a,A^ ± 1.88
Proveget fine (potato proteins)	2.92 ^a,A^ ± 11.26	18.18 ^ab,B^ ± 8.48	4.22 ^a,A^ ± 10.30	4.02 ^a,A^ ± 5.43
Proveget 100 (pea proteins)	6.66 ^a,A^ ± 19.71	22.91 ^ab,B^ ± 6.92	0.83 ^a,AB^ ± 1.40	1.87 ^a,AB^ ± 2.81
Vinigel forte (porcine gelatine)	13.61 ^a,A^ ± 10.77	33.80 ^b,B^ ± 16.89	1.01 ^a,A^ ± 5.31	2.00 ^a,A^ ± 5.14

Different lower-case letters per column in the data indicate significant differences among the different fining tests and different capital letters per lines in the data indicate differences between phenolic families (Tukey’s test, α = 0.05). (Means ± SD, *n* = 3).

**Table 2:** Turbidity reduction (as percentage with respect to the control wine) for the different fining agents.

Fining Agents	% Turbidity Reduction
Bentonite	24.30 ^bc^ *±* 1.55
Proveget fine (potato proteins)	5.16 ^ac^ *±* 0.60
Proveget 100 (pea proteins)	−6.08 ^a^ *±* 0.48
Vinigel Forte (porcine gelatine)	50.25 ^b^ *±* 1.24

Different letters in the data indicate significant differences among the different fining tests (Tukey’s test, α = 0.05). (Means ± SD, *n* = 3).

**Table 3:** Fining indexes for the three cell wall samples in comparison to the commercial fining agents.

Cell Wall Material from Red Grape Skin	Bentonite	Proveget 100 (Pea Proteins)	Proveget Fine (Potato Proteins)	Vinigel Forte (Porcine Gelatine)
Pre-harvest 24º Brix (P24)	48.7 ^a^ ± 11.7	60.7 ^a^ ± 6.6	62.2 ^a^ ± 9.1	62.4 ^a^ ± 3.6
Harvest 24º Brix (H24)	34.9 ^a^ ± 4.6	51.11 ^b^ ± 4.4	51.0 ^b^ ± 4.8	56.2 ^b^ ± 3.2
Over-ripening 24º Brix (O24)	39.9 ^a^ ± 1.5	54.5 ^b^ ± 1.6	55.1 ^b^ ± 1.7	58.3 ^b^ ± 1.7

Result of Equation (1) developed for the fining index (Means ± SD, *n* = 3). Different letters in the data indicate significant differences among indexes for each evaluated cell wall material from red grape skin (Tukey’s test, α = 0.05).

## Data Availability

The original contributions presented in the study are included in the article; further inquiries can be directed to the corresponding author.
